# Supplementary material for: p75NTR Modulation Reduces Oxidative Stress and the Expression of Pro-Inflammatory Mediators in a Cell Model of Rett Syndrome
Source: Biomedicines. 2024 Nov 16;12(11):2624. doi: 10.3390/biomedicines12112624 (PMC11592079; doi:10.3390/biomedicines12112624)
Supplement: Supplementary file 1 [file biomedicines-12-02624-s001.zip › biomedicines-3294215-supplementary.pdf]

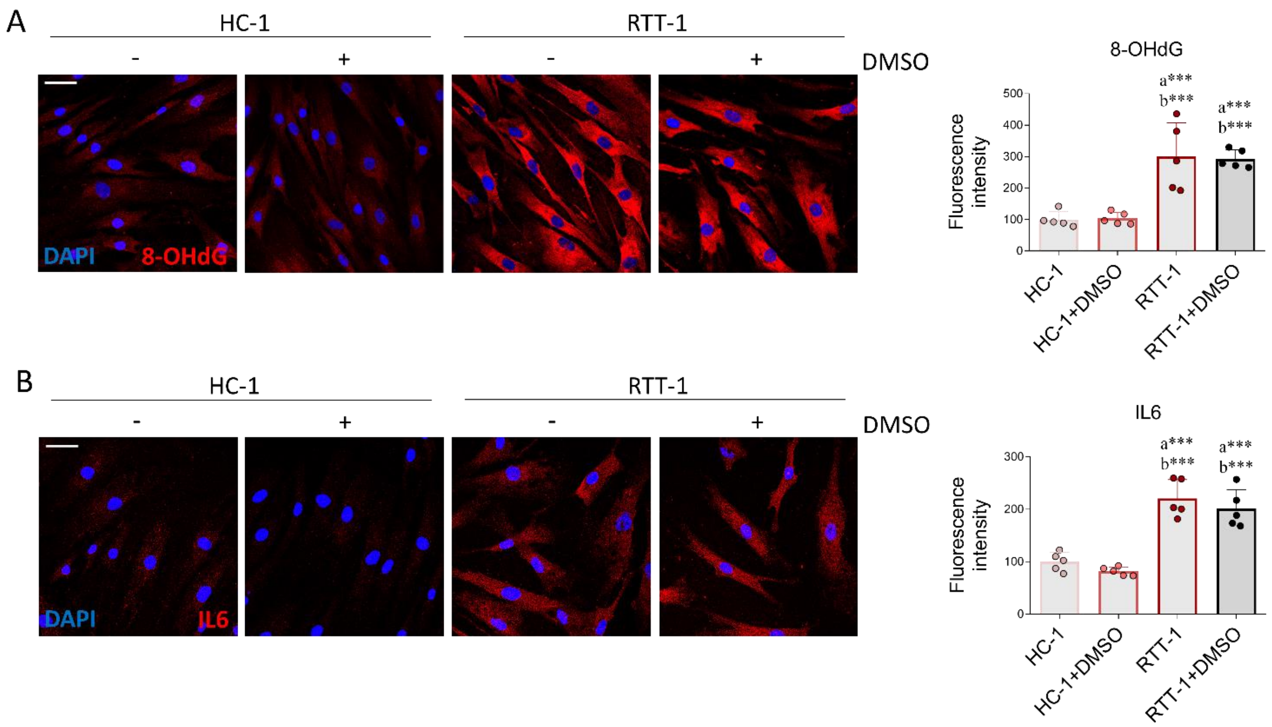

Figure S1. DMSO, added as vehicle at the dose of 0.0001%, affects neither markers of oxidative damage nor the expression of proinflammatory IL6. (**A**) Immunofluorescence and respective quantitative analysis of 8-OHdG in healthy control (HC-1) and Rett (RTT-1) fibroblasts, treated or not with DMSO (0.0001%). After 24 hours, cells were fixed in 4% PFA and stained with antibodies against 8-OHdG (red). DAPI (blue) was employed for nuclear counterstaining. n = 5 biological replicates. (**B**) Immunofluorescence and respective quantitative analysis of IL6 immunoreactivity in HC-1 and RTT-1 cells, treated as in (A). Cells were fixed in 4% PFA and stained with anti-IL6 (red). DAPI (blue) was used to counterstain nuclei. n = 5 biological replicates. Data are expressed as mean ± SD. Statistical analysis was performed by using One-way ANOVA followed by Tukey's post hoc test. Statistical significance is indicated as follows: ***$p < 0.001$. "a" indicates statistical significance vs. HC-1; "b" indicates statistical significance vs HC-1+DMSO. Images were acquired using the Leica TCS SP8 confocal microscope and Leica Application Suite X (LAS X) software at 40X magnification. Scale bar: 50 µm.

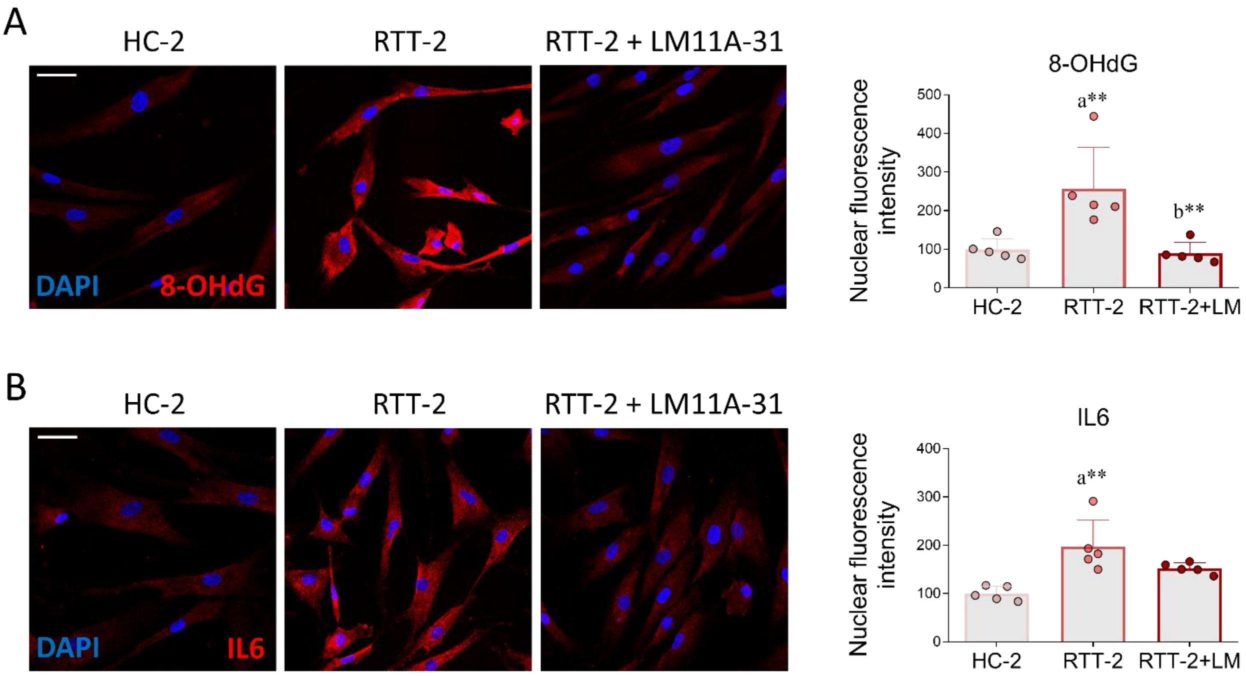

Figure S2. The effects of LM11A-31 on oxidative stress and IL6 are recapitulated in cultured fibroblasts derived from another RTT individual. (**A**) Immunofluorescence and respective quantitative analysis of 8-OHdG in healthy control (HC-2), Rett syndrome fibroblasts (RTT-2) and Rett syndrome fibroblasts treated with LM11A-31 (RTT-2+LM) at the dose of 0.1 µM for 24 hours. Cells were fixed in 4% PFA and stained with antibodies against 8-OHdG (red). DAPI (blue) was employed for nuclear counterstaining. n = 5 biological replicates. (**B**) Immunofluorescence and respective quantitative analysis of IL6 immunoreactivity (red) in HC-2, RTT-2 and RTT-2+LM experimental groups, treated as in (A). DAPI (blue) was used to counterstain nuclei. n = 5 biological replicates. Data are expressed as mean ± SD. Statistical analysis was performed by using One-way ANOVA followed by Tukey's post hoc test. Statistical significance is indicated as follows: $^{**}p < 0.01$. "a" indicates statistical significance vs. HC-2; "b" indicates statistical significance vs HC-2+LM. Images were acquired using the Leica TCS SP8 confocal microscope and Leica Application Suite X (LAS X) software at 40X magnification. Scale bar: 50 µm.
